# The Adsorption of Heavy Metal Ions by Poly (Amidoamine) Dendrimer-Functionalized Nanomaterials: A Review

**DOI:** 10.3390/nano12111831

**Published:** 2022-05-27

**Authors:** Dandan Guo, Shaohua Huang, Yan Zhu

**Affiliations:** 1 Institute of Drug Discovery and Technology, Ningbo University, Ningbo 315211, China; guodandan@nbu.edu.cn; 2 Department of Chemistry, Zhejiang University, Hangzhou 310028, China; 3 Qian Xuesen Collaborative Research Center for Astrochemistry and Space Life Sciences, Ningbo University, Ningbo 315211, China

**Keywords:** adsorbents, poly (amidoamine), heavy metal ions, adsorption mechanisms, further functionalization

## Abstract

Rapid industrialization has resulted in serious heavy metal pollution. The removal of heavy metal ions from solutions is very important for environmental safety and human health. Poly (amidoamine) (PAMAM) dendrimers are artificial macromolecular materials with unique physical and chemical properties. Abundant amide bonds and amino functional groups provide them with a high affinity for heavy metal ions. Herein, PAMAM-functionalized adsorbents are reviewed in terms of different nanomaterial substrates. Approaches in which PAMAM is grafted onto the surfaces of substrates are described in detail. The adsorption isotherms and kinetics of these adsorbents are also discussed. The effects of PAMAM generation, pH, adsorbent dosage, adsorption time, thermodynamics, and ionic strength on adsorption performance are summarized. Adsorption mechanisms and the further functionalization of PAMAM-grafted adsorbents are reviewed. In addition to the positive results, existing problems are also put forward in order to provide a reference for the optimization of PAMAM-grafted adsorbents of heavy metal ions.

## 1. Introduction

With the rapid development of human civilization, environmental pollution caused by industrialization has become a focus of concern all over the world [1,2,3]. Among numerous pollutants, heavy metal ions are some of the most harmful. The main source of heavy metal ion pollution is effluents from industrial activities, such as battery production, electroplating, staining, metallurgy, and rubber production. More than 35 elements could be considered as heavy metals, and most of them are toxic. These heavy metal ions are discharged into the environment with industrial wastewater, enter rivers and soil, and eventually accumulate in the human body through the food chain. Unlike other pollutants, heavy metal ions cannot be metabolized and thus do harm to the human body. Generally, common heavy metal contaminants include Pb, Hg, Cu, Cd, As, Co, Ni, Mn, and Zn [4]. These heavy metal ions interfere with the functions of living creatures by binding to their vital cellular components. For example, excess lead ions can damage the liver, kidneys, central nervous system, and gastrointestinal tract [5,6]. Large amounts of copper ions can cause headache, depression, and learning disorders [7,8]. Respiratory failure, kidney injury, chronic diseases, central nervous system disorders, and brain damage can be caused by mercury [9]. Therefore, the elimination of heavy metal ions from the environment is of great significance to human health.

**Figure 1:**
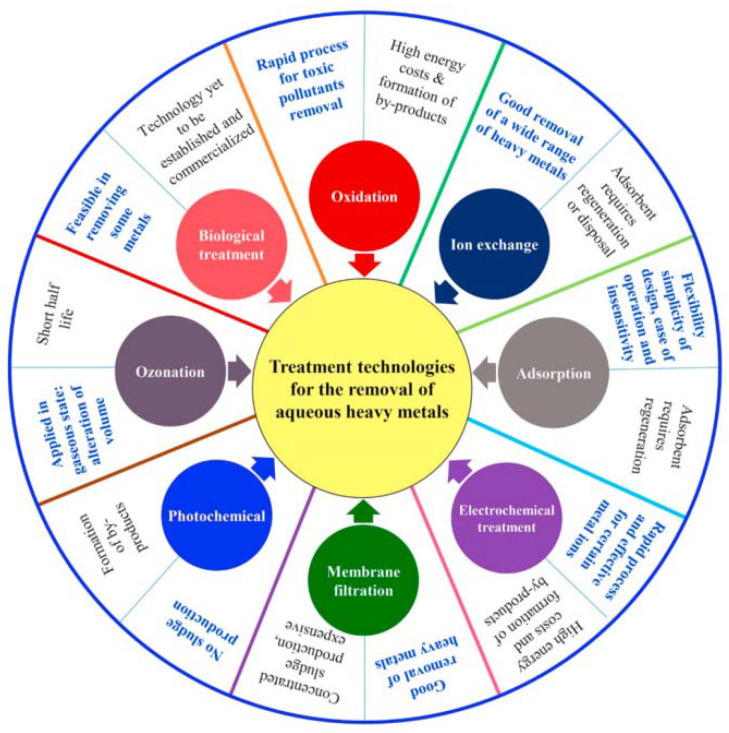
Main characteristics of methods for heavy metal removal. Reprinted with permission from Ref. [28]. 2022, Elsevier.

Considering the harm caused by heavy metal ions, various methods have been applied to remove them from the environment, such as oxidation [10], biological treatments [11], ion exchange [12], electrochemical treatments [13], and adsorption [14,15]. The main characteristics of these methods are shown in Figure 1. Although these methods have been successfully applied in industries, some of them still suffer from unavoidable drawbacks [16,17]. For example, oxidation methods have high energy costs and produce a large amount of byproducts. Biological treatments require a high level of technical skill. Ion exchange resins are poor in thermostability and are difficult to regenerate. Electrochemical treatments consume much electric power and generate secondary waste. According to the existing literature, the adsorption method is outstanding because of its simple process, high adsorption efficiency, and environmentally friendly operation. Therefore, a large number of materials that adsorb heavy metal ions have been developed [18,19,20,21]. 

Over the past few decades, nanomaterial adsorbents have been highly favorable for heavy metal ion removal due to their unique properties, such as their nano size, developed porosity, large specific surface area, high reactivity, strong mechanical properties, hydrophilicity, and dispersibility [22,23]. Generally, these adsorption materials are nano-sized particles consisting of substrates and surface functional groups. Nanomaterial substrates can be divided into inorganic matrixes and organic matrixes, which usually have a large specific surface areas, good stability, and high dispersibility while being easy to modify. Surface functional groups are usually introduced by further functionalization of the substrates, and they always have good affinity for heavy metal ions. Common functional groups include amino groups, carboxyl groups, amide bonds, and sulfhydryl groups [24,25,26,27].

Poly (amidoamine) (PAMAM) dendrimers are synthetic polymer materials with a highly symmetrical structure. They always have controllable molecule chains, vast internal cavities, and abundant functional groups [29]. On the basis of these special structural characteristics, PAMAM dendrimers have many unique properties, such as high hydrophilicity, high dispersity, high bioaffinity, and ease of modification, and thus have been widely used in various fields [30,31,32,33,34]. Especially in adsorbents of heavy metal ions, PAMAM dendrimers play an important role in the modification of nanomaterial substrates [35,36,37,38,39]. A large number of amino and amide functional groups of PAMAM dendrimers can strongly chelate heavy metal ions, thus improving the enrichment efficiency.

**Figure 2:**
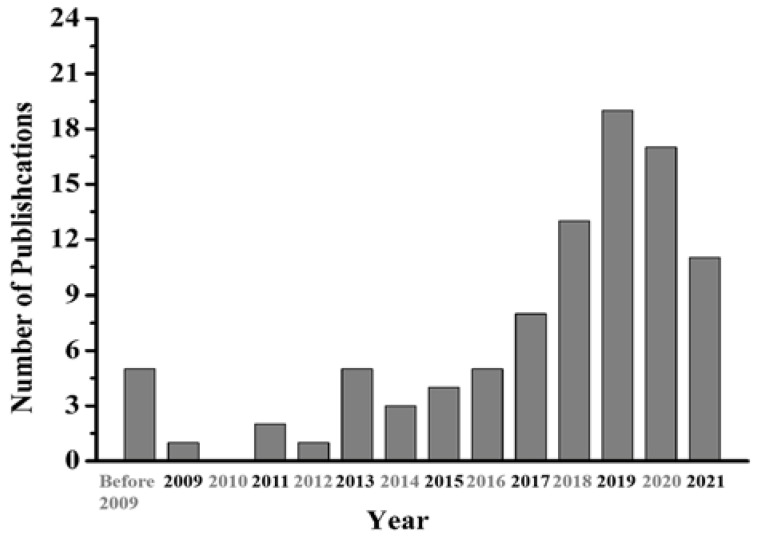
Number of publications involving the PAMAM-grafted adsorbents for heavy metal ions from 2006 to 2022. (Searched from web of science).

Over the past 20 years, PAMAM-functionalized nanomaterials have been reported in nearly 100 papers regarding the adsorption of heavy metal ions (Figure 2), mainly involving silica gel, carbon nanomaterials, magnetic materials, and biopolymers. In addition to the direct grafting of PAMAM dendrimers, the further functionalization of PAMAM-grafted adsorbents has led to increased selectivity and adsorption efficiency. Herein, we review PAMAM-functionalized adsorbents from the perspective of different nanomaterial substrates and discuss the influences of PAMAM generation, pH, adsorbent dosage, adsorption time, thermodynamics, and ionic strength on adsorption efficiency. Adsorption isotherms and adsorption kinetics are also discussed. The adsorption mechanisms and further functionalization of adsorbents are also summarized. Finally, the disadvantages of PAMAM-functionalized adsorbents are explained, which could provide a direction for the future development of PAMAM-grafted materials that adsorb heavy metal ions.

## 2. Chemical and Physical Properties of PAMAM Dendrimers

**Figure 3:**
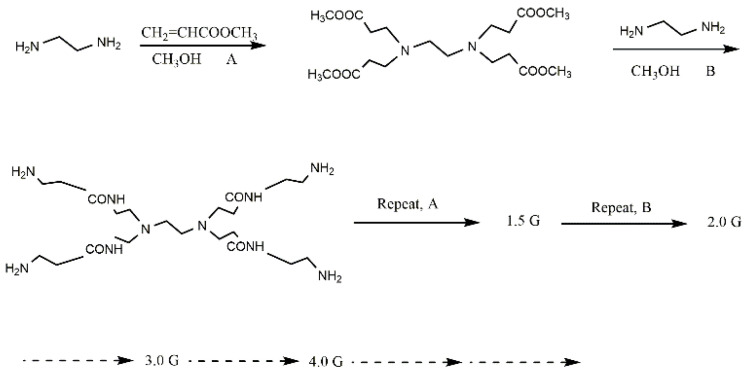
The synthesis procedure of different generations of PAMAM dendrimers. Reprinted with permission from Ref. [42]. 2016, Elsevier.

PAMAM dendrimers are prepared with ethylenediamine as the initial core, which is modified by alternating a Michael addition and an amidation reaction with methyl acrylate and ethylenediamine [40,41]. In this process, the chain length, terminal groups, and molecular size of PAMAM dendrimers can be strictly controlled by varying the number of reaction cycles (Figure 3). The structure generated by each step of the reaction is called a half-generation (0.5 G). Half-generation (0.5 G, 1.5 G, 2.5 G, etc.) PAMAM dendrimers have ester groups as the terminal, and whole-generation (1.0 G, 2.0 G, 3.0 G, etc.) PAMAM dendrimers have amino groups as the terminal. In general, lower-generation (≤3.0 G) PAMAM dendrimers have planar structures, while higher-generation (>4.0 G) PAMAM dendrimers have spatial structures. Moreover, with increasing generations, the molecular chains of PAMAM dendrimers become longer, internal cavities become denser, and the number of functional groups grows exponentially, which therefore provides more binding sites for the adsorption process.

Compared to other traditional materials, PAMAM dendrimers have significant advantages in the adsorption of heavy metal ions: Firstly, PAMAM dendrimers have abundant functional groups. According to the scheme in Figure 3, the number of functional groups increases exponentially with the continuous growth of PAMAM generations. More functional groups in higher generations may provide more binding sites for heavy metal ions. Secondly, PAMAM dendrimers have controllable molecule chains. PAMAM dendrimers are composed of repeated amide structural units generated by repeated and alternating reactions. By varying the reaction cycles, the length of the molecule chains can be effectively regulated, as can the adsorption capacity of PAMAM-grafted adsorbents. The excellent solubility of PAMAM dendrimers improves the dispersion of adsorbents of heavy metal ions, thus leading to higher adsorption efficiency. Finally, PAMAM dendrimers are easily modified, which improves the selectivity and properties of heavy metal ion adsorbents by further derivatization.

## 3. Adsorbents of Different Substrates

As discussed above, PAMAM dendrimers with a large number of internal cavities and amino functional groups are good candidates for the adsorption of heavy metal ions. However, the excellent aqueous solubility of PAMAM dendrimers makes them difficult to separate from solutions. To overcome this problem, many researchers have grafted PAMAM dendrimers to the surfaces of various nanomaterials and obtained satisfactory results. Although the proposed PAMAM-grafted adsorbents could be recovered by centrifugation, filtration, or magnetic fields, their high cost and multistep synthesis make them unsuitable for a wide range of applications. Therefore, the development of more efficient and economical PAMAM-functionalized adsorption nanomaterials is of greater practical significance for the removal of heavy metal ions from aqueous solutions. 

### 3.1. Adsorbents Based on Inorganic Substrates

**Figure 4:**
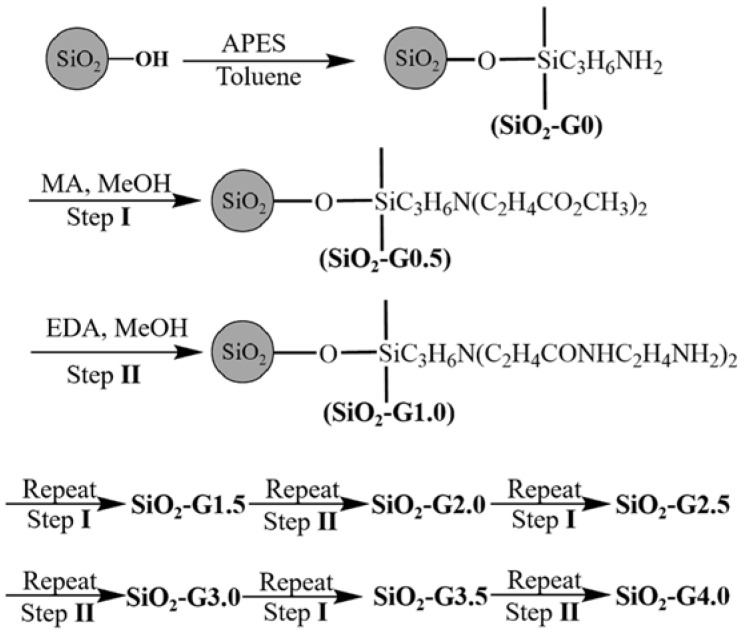
Ideal synthetic routes of ester- and amino-terminated dendrimer-like PAMAM polymers-grafted silica gel. Reprinted with permission from Ref. [43]. 2006, Elsevier.

The first PAMAM-grafted adsorbents for heavy metal ions were proposed with silica gel as the substrate [43]. Silica gel is one of the most widely used adsorbent matrix materials because of its good thermal stability, superior mechanical resistance, modifiability, and high surface area [44,45,46]. Qu et al. prepared a series of ester- and amino-terminated PAMAM-grafted silica gels (SiO_2_-G0-SiO_2_-G4.0) by a divergent method. Firstly, the surface silanol groups were treated with γ–aminopropyltriethoxysilane (APTES) to introduce amino groups to the surface of the silica gel. Afterwards, PAMAM dendrimers were grafted by repeating two processes: (1) the Michael addition of methyl acrylate to surface amino groups; and (2) the amidation of the resulting esters with an ethylenediamine reagent (Figure 4). Further adsorption experiments showed that all of the ester- and amino-terminated dendrimer-like PAMAM-grafted silica gels presented regularities in the adsorption of noble metal ions to a certain extent. On the basis of this work, more researchers applied PAMAM-functionalized silica gels to the adsorption of heavy metal ions and further investigated the adsorption kinetics, the isothermal adsorption model, and the adsorption mechanisms [47,48,49,50,51,52].

In addition to silica gel, other inorganic substrates have also been tested as substrates for PAMAM-grafted adsorbents. For example, Barakat et al. immobilized 4.0G PAMAM dendrimers over titanium(IV) oxide (titania) for the removal of Pb^2+^ from solutions [53]. A maximum Pb^2+^ adsorption was found at pH 7. The adsorption of Pb^2+^ on PAMAM dendrimers conformed to the Langmuir isotherm and the second-order kinetic model. Qin et al. developed a series of (G1.0, G2.0, G3.0, and G4.0) PAMAM-dendrimer-modified attapulgite (ATP) sorbents (G1.0–G4.0 PAMAM-ATP) to remove Hg^2+^ from aqueous solutions [54]. Over 90% of the Hg^2+^ was removed within 80 min at an optimal pH of 5.0. The adsorption was also suitable for the pseudo-second-order kinetic model and the Langmuir model. These inorganic substrates have a large surface area and a high surface active site density but suffer from poor selectivity and biotoxicity. PAMAM grafting can solve these problems effectively and achieve the efficient selective adsorption of heavy metal ions from solutions. 

### 3.2. Adsorbents Based on Biopolymers

In recent years, low-cost adsorbents, such as biopolymers, have attracted increasing attention and have proven to be extremely promising materials [55,56,57]. Polysaccharides are renewable and biodegradable polymers that have great application prospects as an adsorption matrix. A large number of surface functional groups, such as hydroxyl, carboxyl, and amine groups, leads to a high affinity for heavy metal ions. Although polysaccharides have many advantages, including high hydrophilicity, nontoxicity, biodegradability, and environmental friendliness, many problems continue to prevent them from being directly used as adsorbents [58]. Firstly, polysaccharides have poor mechanical strength and have a tendency to agglomerate and form gels in aqueous solutions. Secondly, poor stability in acid solutions greatly limits their applications. Thirdly, the adsorption capacity of most raw polysaccharides for heavy metal ions is not satisfactory because of their low binding site density. Therefore, further chemical modification with PAMAM dendrimers may enhance the physical and chemical properties of polysaccharides and improve the selectivity and efficiency of the adsorption of heavy metal ions. 

Cellulose and chitosan are two of the most abundant polysaccharides and have attracted great interest in relation to the development of PAMAM-grafted adsorbents of heavy metal ions [59,60,61]. Wang et al. proposed PAMAM-functionalized nanocrystalline cellulose (NCC) for Cu^2+^ removal [62]. In their work, amino-functionalized NCC (NCC-NH_2_) was synthesized from raw NCC materials. Subsequently, PAMAM dendrimers were grafted onto the surface of NCC-NH_2_ by two alternate reactions: the Michael addition of amine groups of NCC-NH_2_ to the double bond of methyl acrylate and the amidation of the resulting methyl ester with ethanediamine. The obtained materials were applied as adsorbents in the removal of Cu^2+^ from water solutions. Compared with raw NCC materials, PAMAM-functionalized NCCs showed a significantly higher adsorption capacity. The adsorption isotherm of Cu^2+^ by PAMAM-grafted NCC follows the Langmuir model. Similarly, Zarghami et al. designed a chitosan-PAMAM dendrimer biosorbent for heavy metal removal and studied its adsorption kinetics and thermodynamics [63]. Adsorption experiments showed that the adsorption capacity of as-prepared adsorbents with 3.0G PAMAM is 18 times higher than that of materials with chitosan only. Thermodynamic and kinetic studies showed that the adsorption of Pb^2+^ conforms to the Langmuir isotherm model and the pseudo-second-order kinetic model. 

In addition to polysaccharides, biochar is another novel biopolymer that has been garnering increasing attention for the elimination of heavy metal ions from water. Generally, biochar is synthesized by a thermochemical decomposition process of biomass under oxygen-limited conditions. The obtained material has unique advantages, such as an easy preparation process, a high cation exchange capacity, and low costs and environmental impacts. However, a low specific surface area limits its removal efficiency. To overcome this issue, Yin et al. modified magnetic biochar with PAMAM dendrimers [64]. With abundant—NH_2_ groups on the surface, the obtained materials showed outstanding performances in Cu^2+^ adsorption. The maximal adsorption amount was 251.81 mg g^−1^. A batch adsorption experiment proved that the adsorption process of Cu^2+^ was better fitted with the Langmuir isotherm model and the pseudo-second-order model.

### 3.3. Adsorbents Based on Carbon Nanomaterials

Carbon nanomaterials are one of the most widely used types of nanomaterials in the field of materials and chemistry [65,66,67,68]. They have many unique characteristics, such as good chemical stability, a high surface area, satisfactory flexibility, and electrical conductivity. On the basis of these properties, carbon nanomaterials have played an important role in various fields. In analytical chemistry, carbon nanomaterial adsorbents have also shown excellent performance in sample pretreatment [69,70]. Due to their limited surface functional groups, these adsorbents were always synthesized with raw carbon nanomaterials as substrates and then modified with various functional molecules. Among these adsorbents, PAMAM-grafted carbon nanomaterials were good candidates for the adsorption of heavy metal ions. 

**Figure 5:**
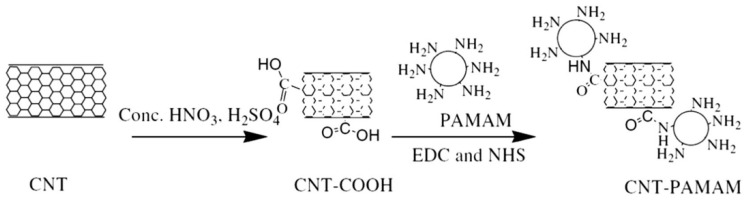
Synthesis process of PAMAM/CNT nanocomposite. Reprinted with permission from Ref. [71]. 2016, Elsevier.

In 2016, Hayati et al. proposed a PAMAM/CNT nanocomposite as a super-capacity adsorbent for heavy metal ions from wastewater [71]. Carbon nanotubes (CNTs) were oxidized to carboxylic acid functional groups (CNT-COOH) in a concentrated mixture of H_2_SO_4_ and HNO_3_. After that, PAMAM dendrimers were grafted to the surface of CNTs by reaction with surface carboxyl groups (Figure 5). Finally, the adsorption capacities of the PAMAM/CNT nanocomposites for Ni^2+^, Zn^2+^, As^3+^, and Co^2+^ were examined. Maximum adsorption was obtained at pH = 8. The results showed that the Langmuir isotherm and the pseudo-second-order kinetic model are the most appropriate models for the adsorption of heavy metal ions. 

In addition to carbon nanotubes, graphene and graphene oxide are also important carbon nanomaterials. Peer et al. synthesized PAMAM-grafted magnetic graphene oxide nanosheets and investigated their abilities to effectively remove Cd^2+^, Pb^2+^, and Cu^2+^ from an aqueous solution [72]. The results of the batch adsorption experiment showed that the adsorption capacities for Cd^2+^, Pb^2+^, and Cu^2+^ were 435.85, 326.729, and 353.59 mg g^−1^, respectively. The obtained data were well-fitted with the Freundlich isotherm model and the pseudo-second-order kinetic model. Kommu et al. conducted molecular dynamics simulations to understand the effect of PAMAM-grafted graphenes (GS) and graphene oxide (GO) on the adsorption properties of heavy metal ions [73]. Using Pb^2+^ as an example: the adsorption capacity of the Pb^2+^ ion was calculated in the following order: GO-PAMAM-COO- > GO-PAMAM-OH > GO-PAMAM > GO > GS-PAMAM-COO- > GS-PAMAM-OH > GS-PAMAM > GS. The adsorption behaviors were well described by the Langmuir isotherm model. It should be mentioned that the interaction between Pb^2+^ and PAMAM dendrimers was shown to play a significant role in adsorption, indicating the importance of functional PAMAM dendrimers for the adsorption of heavy metal ions. 

### 3.4. Adsorbents Based on Membrane Materials

With the rapid development of bioscience and the pharmaceutical industry, membrane separation has been widely used as an important purification technology [74,75,76]. Biomacromolecules are increasingly being purified by membrane separation [77,78]. In addition, membrane separation technology is important for water treatment due to its ease of operation, its high adsorption efficiency, and its reusability [79,80]. Generally, polyvinylidene fluoride (PDVF) is the most widely used membrane material due to its excellent mechanical strength, thermal stability, acid and alkali resistance, and low cost [81]. However, PDVF membrane materials have few functional groups on their surfaces, which is unfavorable to the adsorption of heavy metal ions. Therefore, further modifications are needed to improve their adsorption ability.

**Figure 6:**
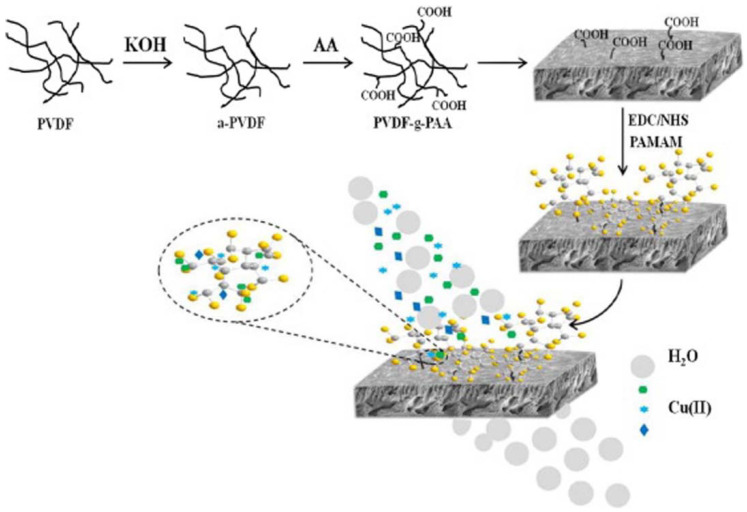
Scheme of preparation of PVDF-g-PAA-PAMAM membrane. Reprinted with permission from Ref. [84]. 2019, Elsevier.

Considering their high-affinity interaction with heavy metal ions, PAMAM dendrimers were used to modify membrane materials and improved their adsorption capacity [82,83,84,85]. With PDVF as a raw substrate, Kotte et al. reported a one-pot method for the preparation of a new family of mixed matrix PVDF membranes with in situ synthesized PAMAM dendrimers [83]. The obtained materials have neutral and hydrophilic surface layers, a high load of PAMAM dendrimers, and strong mechanical integrity. Further experiments showed that the PAMAM-functionalized PVDF membranes have a high adsorption capacity for Cu^2+^. Recently, Sun et al. also proposed a novel kind of PVDF-g-PAA-PAMAM membrane by grafting different-generation PAMAM dendrimers onto PVDF-g-PAA membranes [84]. The membrane materials were first modified with acrylic acid, and a large number of carboxyl groups were introduced to the surface. Subsequently, PAMAM dendrimers were grafted onto membrane materials by a dehydration reaction with surface carboxyl groups (Figure 6). As the grafting of PAMAM dendrimers can greatly improve the water flux and the affinity for heavy metal ions, the adsorption capacity of the obtained PVDF-g-PAA-PAMAM membranes for Cu^2+^ improved from 2.6 mg/g to 100.98 mg/g.

### 3.5. Adsorbents in Gel State

**Figure 7:**
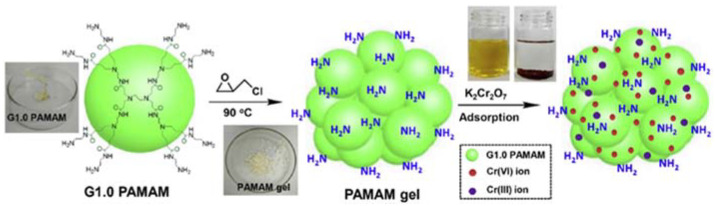
Schematic illustration of the preparation of amine-rich PAMAM gel from G1.0 PAMAM for removal of toxic Cr(VI) ions from water. Reprinted with permission from Ref. [86]. 2019, Elsevier.

PAMAM dendrimers are a kind of artificial macromolecular material with multiple terminal functional groups, which can be cross-linked to form network structures. Zhou et al. prepared an amine-rich PAMAM gel in a single step based on the cross-linking reaction between PAMAM and epichlorohydrin (Figure 7) [86]. The obtained PAMAM gel possesses abundant amine groups, a positively charged surface, a porous microstructure, and a high swelling ratio, which helps it to adsorb Cr(VI) ions from water solutions. The adsorption process could be well-fitted to the pseudo-second-order kinetic model and the Langmuir isotherm. The PAMAM gel presented favorable reusability after five cycles of desorption–adsorption. Recently, i-carrageenan/PAMAM aerogels and gelatin/PAMAM aerogels were also created by cross-linking PAMAM dendrimers with i-carrageenan and gelatin, respectively [87,88]. The obtained aerogels showed high adsorption efficiencies for Cr(VI), Mn(II), Co(II), Cu(II), and Cd(II). This work will provide instruction for the development of PAMAM-grafted materials for heavy metal adsorption with lower cost and higher efficiency.

## 4. Adsorption Studies

### 4.1. Influence of PAMAM Generations

PAMAM dendrimers were synthesized with ethylenediamine as an initial core, which was modified by alternating Michael additions and amidation reactions. Each repeat unit in PAMAM was called a generation (G). Each half-generation PAMAM dendrimer terminates with ester groups, and whole-generation PAMAM dendrimers terminate with amino groups. Moreover, the number of terminal functional groups increases exponentially with increasing PAMAM generations. Therefore, the grafting of PAMAM dendrimers of different generations may have different effects on the adsorption efficiency of heavy metal ions. 

To verify this conjecture, Fu et al. investigated the adsorption of Mn^2+^ from aqueous solutions by silica-gel-grafted PAMAM dendrimers in different generations (SiO_2_-G0~SiO_2_-G4.0) [89]. The adsorption results showed that the adsorption capacity of amino-terminated PAMAM dendrimers was higher than that of the corresponding ester-terminated ones, which could be interpreted as meaning that amino groups display a stronger ability to bind to heavy metal ions. The adsorption capacity of the adsorbents grafted with ester-terminated PAMAM dendrimers follows the order of SiO_2_-G3.5 > SiO_2_-G2.5 > SiO_2_-G1.5 > SiO_2_-G0.5. With increasing generations PAMAM dendrimers, more functional groups were grafted to the surface of SiO_2_, leading to a higher adsorption efficiency. However, the adsorption capacity of the adsorbents grafted with amino-terminated PAMAM dendrimers follows the order of SiO_2_-G3.0 > SiO_2_-G2.0 > SiO_2_-G4.0 > SiO_2_-G1.0. In PAMAM generations varying from 1.0G to 3.0G, adsorption efficiency increased with PAMAM generations. The adsorption capacity of SiO_2_-G4.0 is lower than that of SiO_2_-G2.0 and SiO_2_-G3.0, implying that a high generation of PAMAM grafted onto the surface of SiO_2_ did not mean a high adsorption capacity. A high degree of cross-linking and steric hindrance in PAMAM of higher generations may hinder the interaction between heavy metal ions and PAMAM dendrimers, thus reducing the adsorption efficiency [38,90]. 

In addition to the PAMAM structure, solvents also affect the adsorption capacity of adsorbents in different PAMAM generations. For example, the order of adsorption capacity of SiO_2_-G1.0, SiO_2_-G2.0, and SiO_2_-G3.0 for Cu^2+^ was SiO_2_-G1.0 > SiO_2_-G2.0 > SiO_2_-G3.0 in an ethanol solution, which is different from that of SiO_2_-G2.0 > SiO_2_-G3.0 > SiO_2_-G1.0 in an aqueous solution [44]. Similarly, the order of the adsorption capacity for an Hg^2+^ aqueous solution was SiO_2_-G1.0 < SiO_2_-G2.0 < SiO_2_-G3.0, which is quite different from the adsorption in an ethanol solution (SiO_2_-G2.0 > SiO_2_-G1.0 > SiO_2_-G3.0) [91]. The differences between adsorption capabilities in different solvents may be related to the solvated structure of heavy metal ions. 

### 4.2. Influence of pH

In the process of the adsorption of heavy metal ions, the pH of a solution is a key factor affecting the adsorption results, as it affects not only the structure of surface functional groups of adsorbents but also the state of heavy metal ions. Therefore, the influence of pH on PAMAM-grafted adsorbents of heavy metal ions has also been investigated by many researchers.

According to the adsorption results of a variety of heavy metal ions, PAMAM-grafted adsorbents showed the highest adsorption efficiency under neutral to weakly acidic conditions (pH = 5–7) [71,92,93,94]. PAMAM dendrimers have primary amine groups with a pKa value of 6.85 and internal tertiary amine groups with a pKa value of 3.86 [95]. Most amino groups are protonated at lower pH values, leading to strong electrical repulsion with heavy metal cations which hinders adsorption interactions. In addition, a large amount of H^+^ in an acidic solution will compete for adsorption sites with heavy metal ions. As the pH increases, the amino groups gradually deprotonate, and the adsorption capacity for heavy metal ions is enhanced. As the pH further increases, heavy metal ions will be hydrolyzed. Therefore, most PAMAM-functionalized adsorbents exhibit their largest adsorption capacities at pH 5–7. 

### 4.3. Influence of Other Parameters

Considering the special structure of PAMAM dendrimers, PAMAM generations and pH have exhibited great impacts on PAMAM-functionalized adsorbents. However, other parameters that affect the adsorption efficiency are also worth discussing, such as adsorbent dosage, adsorption time, thermodynamics, and ionic strength.

To investigate the effect of adsorption dosage, Peer et al. [72] applied 2–10 mg/100 mL PAMAM-grafted magnetic graphene oxide nanosheets (mGO2nd-PAMAM) for Cd^2+^, Pb^2+^, and Cu^2+^ adsorption, respectively. The results showed that adsorption efficiency for heavy metal ions increased with increasing adsorbent dosages. It could be that a higher amount of adsorbent provided more active sites and trapped more heavy metal ions on the adsorbent sites. It is worth mentioning that the adsorption amount of the unit adsorbent decreased at a certain quantity of the adsorbent, causing aggregation of the adsorbent and possibly decreased availability of active sites. In addition to adsorption dosage, they also evaluated the influence of adsorption time. Appropriate amounts of mGO2nd-PAMAM adsorbents were dispersed in 100 mL of 30 mg L^−1^ of Cd^2+^ at pH 7 and 20 mg L^−1^ Pb^2+^ and Cu^2+^ at pH 6 and 7, respectively. According to the results, the removal efficiency of the heavy metal ions increased sharply within the first 10 min and finally reached equilibrium. The adsorption equilibrium time of Cd^2+^, Pb^2+^, and Cu^2+^ were 120 min, 100 min, and 90 min, respectively, which implies that PAMAM-functionalized adsorbents exhibit different adsorption times for different heavy metal ions.

Temperature is considered one of the most important factors in adsorption processes, which may affect the adsorption efficiency of heavy metal ions. Fu et al. [89] evaluated the adsorption thermodynamics of Mn^2+^ on PAMAM-grafted silica gel particles. Adsorption experiments at three different temperatures (288, 298, and 308 K) with four different adsorbents (SiO_2_-G1.0, SiO_2_-G2.0, SiO_2_-G3.0, and SiO_2_-G4.0) were performed. The results were calculated by the Gibbs free energy change (ΔG, kJ/mol), enthalpy change (ΔH, kJ/mol), and entropy change (ΔS, J/mol K). The calculated parameters are summarized in Table 1. The negative values of ΔG indicated that the adsorption of heavy metal ions was spontaneous. The positive values of ΔH revealed that the adsorption was endothermic. The positive values of ΔS implied increasing disorder at the solid–liquid interface.

In the practical application of adsorbents of heavy metal ions, the presence of other ions in the solution may interfere with the adsorption efficiency. Therefore, Yin et al. [96] proposed silica-gel/PAMAM dendrimer hybrid adsorbents and discussed the effects of ionic strength on Cr^3+^ adsorption. The effects of ionic strength on the adsorption were evaluated by introducing Na^+^, Ca^2+^, and Mg^2+^ into the solution. Compared to the saturated adsorption capacity, the adsorption capacity for Cr^3+^ decreased with the interference of other ions. The reason for the decrease of the adsorption capacity could be attributed to the competition between Na^+^, Ca^2+^, Mg^2+^, and Cr^3+^ for the available adsorption sites. Coincidentally, the effects of ionic strength were also discussed by Liu et al. [97], who proposed a PAMAM-based dithiocarbamate magnetic composite for the adsorption of Co^2+^ from an aqueous solution. They synthesized a PAMAM-grafted magnetic composite and further derived PAMAM terminal groups with a carbon disulfide reagent. The obtained adsorbents exhibited good adsorption effects on Co^2+^ in the presence of interfering ions, indicating that further derivatization of PAMAM-functionalized adsorbents could bypass the effect of ionic strength and improve selectivity for the adsorption of heavy metal ions.

Considering the influence of adsorption conditions, the adsorption parameters of PAMAM-functionalized adsorbents of heavy metal ions were fully optimized in the experimental process in order to obtain the maximum adsorption efficiency. The optimized parameters for different PAMAM-functionalized nanomaterials are shown in Table 2. According to the table, most adsorbents exhibited a maximum sorption capacity at 298 K with the pH at 5–7. Adsorption equilibrium time varied from 30 min to 24 h. With the optimized conditions, most of the adsorption processes complied with the Langmuir isotherm model and the pseudo-second-order kinetic model.

## 5. Adsorption Mechanisms

According to the structure of PAMAM dendrimers, the possible binding sites for heavy metal ions included a core tertiary amine, interior amides, and terminal primary amines. To explore the potential adsorption mechanisms, X-ray photoelectron spectroscopy (XPS) [99,100] and density functional theory (DFT) calculations [50,101,102] were performed.

**Figure 8:**
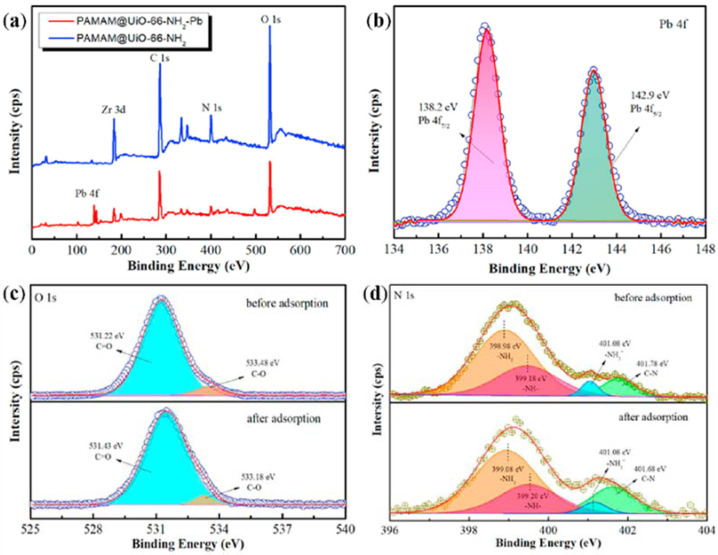
XPS total spectra (**a**), Pb 4f after adsorption (**b**), N 1s before adsorption and after adsorption (**c**), O 1s before adsorption and after adsorption (**d**). Reprinted with permission from Ref. [103]. 2021, Elsevier.

Recently, Ji et al., proposed PAMAM@UiO-66-NH_2_ nanocomposites for the selective removal of Pb^2+^ [103]. In their work, XPS was used to investigate the adsorption mechanism for Pb^2+^. The PAMAM@UiO-66-NH_2_ adsorbents before and after Pb^2+^ adsorption were characterized by XPS. The obtained spectra are displayed in Figure 8. Compared to raw PAMAM@UiO-66-NH_2_, a new peak of Pb 4f was observed in the used adsorbents, which confirm the adsorption of Pb^2+^. The O 1s and N 1s spectra before and after adsorption were further compared. The binding energy peaks of C=O (531.22 eV), C-O (533.48 eV), -NH_2_ (398.98 eV), and -NH- (399.18 eV) shifted to 531.44, 533.18, 399.08, and 399.20 eV, respectively. These results proved that both the O groups and the N groups undergo complexation with Pb^2+^. 

**Figure 9:**
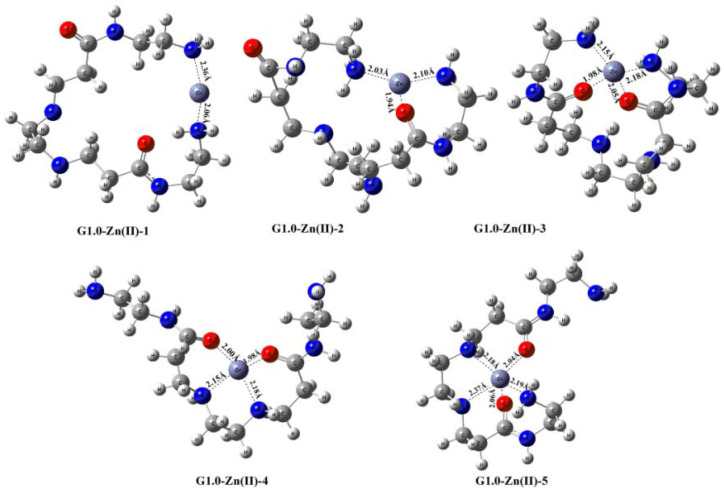
Optimized coordination structures of 1.0G PAMAM with Zn(II). Reprinted with permission from Ref. [90]. 2020, Elsevier.

In addition to XPS, DFT calculations were applied to find optimal configurations between heavy metal species and PAMAM dendrimers, which helps facilitate further study of the adsorption mechanisms. For example, Wu et al. revealed the adsorption mechanism of Zn(II) on 1.0G PAMAM-grafted silica gel (G1.0-SG) [90]. Five possible coordination geometries formed between 1.0G PAMAM and Zn(II) may appear during the removal of Zn(II) (Figure 9). The binding energies (BE, absolute value) for Zn of different coordination modes were found to conform to the trend of G1.0-Zn(II)-1 < G1.0-Zn(II)-2 < G1.0-Zn(II)-3 < G1.0-Zn(II)-4 < G1.0-Zn(II)-5, indicating that the chelation between primary and second amino groups with Zn(II) dominates the adsorption, and the formation of pentacoordinate chelates dominates the uptake of Zn(II) by G1.0-SG. 

## 6. Further Functionalization of PAMAM-Grafted Adsorbents

According to the published literature, PAMAM-grafted adsorbents based on various nanomaterials have shown good stability, high efficiency, and recyclability in the adsorption of heavy metal ions. However, the selectivity of most PAMAM-grafted adsorbents must be improved. As a remedy, PAMAM-grafted adsorbents were further functionalized with special functional groups to improve their adsorption efficiency and selectivity.

According to the hard−soft acid−base (HSAB) theory, sulfur-containing ligands display a strong affinity and adsorption selectivity toward Hg^2+^ [104]. Therefore, various methods have been proposed to graft sulfur-containing functional groups onto the terminals of PAMAM-functionalized adsorbents. For example, Niu et al. synthesized silica gel-supported sulfur-capped PAMAM dendrimers by reacting PAMAM-grafted SiO_2_ adsorbents with methyl isothiocyanate [49]. Ghodsi et al. proposed a dendrimer-functionalized magnetic nano-sorbent and further modified sulfur-containing groups on the terminal PAMAM dendrimers with a 2-thiophene carbonyl chloride reagent [105]. Due to the remarkable binding ability between sulfur-containing ligands and Hg^2+^, all of these functionalized adsorbents showed a high adsorption capacity and selectivity for Hg^2+^. These adsorption processes were well-fitted with the pseudo-second-order kinetic model and the Langmuir isotherm model. In addition to sulfur-containing ligands, PAMAM-grafted adsorbents were also functionalized with EDTA [106], P,P-dichlorophenylphosphine oxide [107], chloroacetic acid [108,109], diglycolamic acid [39,110], and Schiff base [111]. Further surface modification of PAMAM-grafted adsorbents introduced more chelating groups and negative surface charges, which amplified their combination ability with heavy metal ions and improved their adsorption properties.

## 7. Conclusions and Outlook

In this review, PAMAM-grafted nanomaterials for heavy metal ion removal were examined. Over the past few decades, most adsorbents have been synthesized with silica gel, metal oxides, biopolymers, carbon nanomaterials, and membranes as substrates. PAMAM dendrimers have been grafted onto substrates by alternating Michael additions and amidation reactions with methyl acrylate and ethylenediamine. At the same time, PAMAM-cross-linked adsorbents in a gel state have also been proposed. All of these adsorbents showed satisfactory performance in the adsorption of heavy metal ions.

For the purposes of further research, the adsorption isotherms and kinetics of these adsorbents have been discussed. The effects of PAMAM generation, pH, adsorbent dosage, adsorption time, thermodynamics, and ionic strength on adsorption efficiency and the adsorption mechanism have also been discussed. Generally, most PAMAM-functionalized adsorbents followed the Langmuir isotherm model and the pseudo-second-order kinetic model, indicating that heavy metal ions undergo single-layer adsorption on the adsorbent surface and that the adsorption rate is controlled by the chemical adsorption mechanism. Whole-generation PAMAM dendrimers exhibit better adsorption efficiency than the corresponding half-generation PAMAM dendrimers. With increasing PAMAM generations, the number of surface functional groups increases, and the adsorbents have a higher adsorption capacity. However, this regularity will disappear after 4.0 G PAMAM dendrimers, which could indicate a high degree of cross-linking and steric hindrance. Most PAMAM dendrimers have shown the best adsorption results at pH 5–7, since neither protonation of the PAMAM amino groups nor hydrolysis of heavy metal ions have been observed in this range. Adsorbent dosage and ionic strength will affect the adsorption efficiency to a certain extent. PAMAM-functionalized adsorbents have exhibited different adsorption times for different heavy metal ions. The adsorption process is spontaneous and endothermic. In addition, the adsorption mechanisms of PAMAM-grafted adsorbents have been investigated via XPS and DFT calculations. Both O groups and N groups undergo complexation with heavy metal ions. The chelation between primary and secondary amino groups dominates the adsorption. Finally, several studies have focused on the further functionalization of PAMAM-grafted adsorbents to obtain higher selectivity and adsorption properties. 

The positive results of these investigations will support the future applications of PAMAM-grafted adsorbents of heavy metal ions. However, the adsorption of heavy metal ions can be further improved. With the rapid development of materials, more high-performance porous materials can be used as novel substrates for PAMAM adsorbents to improve PAMAM grafting efficiency, as well as the adsorption efficiency of heavy metal ions. In addition, the steric hindrance of high-generation PAMAM dendrimers needs to be addressed by novel assembly methods. Thirdly, further functional modifications of PAMAM-grafted adsorbents are in the preliminary stage, and more research will provide broader application prospects for PAMAM-grafted materials that adsorb heavy metals.

## Figures and Tables

**Table 1:** Thermodynamic parameters for the adsorption of Mn^2+^ [89]. Reprinted with permission from Ref. [89]. 2019, Elsevier.

Adsorbents	T (K)	ΔG (kJ mol^−1^)	ΔH (kJ mol^−1^)	ΔS (J mol^−1^ K^−1^)
SiO_2_-G1.0	288	−11.45	89.83	351.65
	298	−14.96		
	308	−18.48		
SiO_2_-G2.0	288	−12.65	59.96	252.12
	298	−15.17		
	308	−17.69		
SiO_2_-G3.0	288	−15.62	6.13	75.56
	298	−16.38		
	308	−17.13		
SiO_2_-G4.0	288	−13.98	40.36	188.68
	298	−15.87		
	308	−17.75		

**Table 2:** Adsorption of heavy metal ions by different PAMAM functionalized nanomaterials.

Substrates	Target Ions	Optimal Parameters	Maximum Sorption Capacity	Adsorption Kinetics	Adsorption Isotherms	Ref.
Silical gel	Fe^3+^	Dosage: 0.05 g/20 mL Time: 24 h Temperature: 278 K	0.58 mmol/g	Pseudo-second-order	Langmuir	[98]
Titania	Pb^2+^	Dosage: 0.5 g/L pH: 7 Time: 60 min Temperature: 298 K	400 mg/g	Pseudo-second-order	Langmuir	[53]
Cellulose	Cu^2+^	Dosage: 200 mg/L pH: 5.5 Time: 24 h Temperature: 29 8K	92.07 mg/g	N.D.	Langmuir	[62]
Chitosan	Pb^2+^	Dosage: 0.01 g/mL pH: 6 Time: 24 h Temperature: 298 k	58.5 mg/g	Pseudo-second-order	Langmuir	[63]
Biochar	Cu^2+^	pH: 2.0–8.0 Time: 24 h Temperature: 318 K	251.81 mg/g	Pseudo-second-order	Langmuir	[64]
Carbon nanotubes	Ni^2+^ Zn^2+^ As^3+^ Co^2+^	Dosage: 0.03 g/L pH = 8 Time: 30 min Temperature: 298 K	3350–3900 mg/g	Pseudo-second-order	Langmuir	[71]
magnetic graphene oxide	Cd^2+^ Pb^2+^ Cu^2+^	Dosage:5–6 mg/100 mL pH: 6–7 Time: 90–120 min Temperature: 298 K	326.729–435.85 mg/g	Pseudo-second-order	Freundlich	[72]
PVDF-g-PAA-PAMAM membrane	Cu^2+^	Time: 110 min Temperature: 298 K	100.98 mg/g	Lagergren second-order	N.D. ^1^	[84]
PAMAM gel	Cr (VI)	Dosage: 50 mg/10 mL pH: 2 Time: 60 min Temperature: 293 K	267.4 mg/g	Pseudo-second-order	Langmuir	[86]

^1^ Not detected.

## Data Availability

Not applicable.
